# Mutual promotion of co-condensation of KRAS G-quadruplex and a well-folded protein HMGB1

**DOI:** 10.1093/nar/gkad938

**Published:** 2023-10-28

**Authors:** Yu Wang, Kaiming Cao, Mingxi Zong, Siming Yuan, Na Zhang, Yangzhong Liu

**Affiliations:** Department of Pharmacy, the First Affiliated Hospital of USTC, Division of Life Sciences and Medicine; Department of Chemistry, University of Science and Technology of China, Hefei, Anhui 230001, China; Department of Pharmacy, the First Affiliated Hospital of USTC, Division of Life Sciences and Medicine; Department of Chemistry, University of Science and Technology of China, Hefei, Anhui 230001, China; School of Medicine, Xiamen University, Xiamen, Fujian 361102, China; Department of Pharmacy, the First Affiliated Hospital of USTC, Division of Life Sciences and Medicine; Department of Chemistry, University of Science and Technology of China, Hefei, Anhui 230001, China; High Magnetic Field Laboratory, Chinese Academy of Sciences, Hefei 230031, China; Department of Pharmacy, the First Affiliated Hospital of USTC, Division of Life Sciences and Medicine; Department of Chemistry, University of Science and Technology of China, Hefei, Anhui 230001, China

## Abstract

Liquid−liquid phase separation (LLPS) of G-quadruplex (GQ) is involved in many crucial cellular processes, while the quadruplex-folding and their functions are typically modulated by specific DNA-binding proteins. However, the regulatory mechanism of binding proteins, particularly the well-folded proteins, on the LLPS of GQs is largely unknown. Here, we investigated the effect of HMGB1 on the condensation of a G-quadruplex of KRAS promoter (GQ^KRAS^). The results show that these two rigid macro-biomolecules undergo co-condensation through a mutual promotion manner, while neither of them can form LLPS alone. Fluidity measurements confirm that the liquid-like droplets are highly dynamic. HMGB1 facilitates and stabilizes the quadruplex folding of GQ^KRAS^, and this process enhances their co-condensation. The KRAS promoter DNA retains quadruplex folding in the droplets; interference with the GQ-folding disrupts the co-condensation of GQ^KRAS^/HMGB1. Mechanistic studies reveal that electrostatic interaction is a key driving force of the interaction and co-condensation of GQ^KRAS^/HMGB1; meanwhile, the recognition of two macro-biomolecules plays a crucial role in this process. This result indicates that the phase separation of GQs can be modulated by DNA binding proteins, and this process could also be an efficient way to recruit specific DNA binding proteins.

## Graphical Abstract

**Graphical Abstract:**
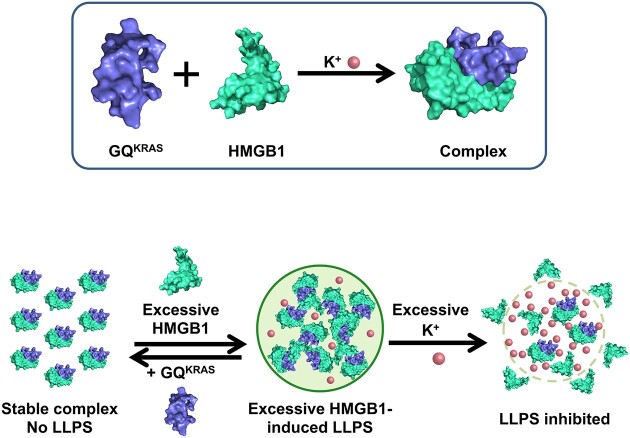

## Introduction

Liquid − liquid phase separation (LLPS) is a ubiquitous mechanism that regulates intracellular activities by assembling diverse macro-biomolecules through multivalent interactions (1–3). In addition to the formation of membraneless organelles, the co-condensation of various biological molecules, such as proteins and DNA, is involved in many biochemical processes, including gene transcription, DNA repair and cellular signaling (2–5). The reversibility of the formation of condensates plays pivotal roles in the regulation of functions of a variety of biomacromolecules involved in LLPS (6,7); hence, it is of considerable importance to understand the modulation of phase transition processes.

G-quadruplex (GQ) is a noncanonical structure of nucleic acids formed by guanine-rich DNA and RNA sequences through Hoogsteen hydrogen bonding. GQs are often found in oncogene promoters and telomeres under physiological conditions, playing essential roles in the regulation of gene transcription and telomere function. Dysregulation of GQ folding could lead to tumorigenesis and neurodegenerative disorders (8,9). Recently, GQ was found to be involved in LLPS of nucleic acids, and perturbation of the GQ folding mitigates their phase transition (10–17). Nevertheless, the GQ folding of nucleic acids and their functions are typically regulated by various DNA binding proteins, (18) and the knowledge about LLPS on the protein regulation of GQ DNA is very limited. Hence, exploration of the co-condensation of GQ with proteins is highly desired.

KRAS is a well-known oncogene and its promoter contains multiple G-rich sequences that can form a GQ structure. The transcription of the KRAS gene can be regulated by the folding of GQ in the promoter sequence; (19,20) therefore, this G-rich repeat is proposed as a therapeutic target for modulating KRAS expression (21–24). Structural investigation indicated that a 22-mer sequence within the promoter sequence, termed 22R, adopts a parallel GQ structure (Supplementary Figure S1A) (22). On the other hand, HMGB1 is an architectural nuclear protein that can bind to DNA and regulate multiple genomic processes (25). A recent study showed that HMGB1 can interact with the KRAS promoter sequence and stabilize its GQ conformation (26).

A recent study showed that the formation of the GQ structure promotes the LLPS of histone H1 (27). Histone H1 contains large IDRs, which is a key factor facilitating LLPS of the protein with DNA (28). However, many DNA binding proteins possess well-folded structures and have low LLPS tendency. It is unknown whether the folded DNA-binding proteins could co-condensate with GQ DNA and regulate the function in the condensates. In this work, we investigated the LLPS of HMGB1 and the KRAS promoter DNA (22R) in its GQ structure (termed GQ^KRAS^). Interestingly, the result reveals that HMGB1 undergoes co-condensation with GQ DNA in a mutual promotion manner.

## Materials and methods

### Oligonucleotides

The DNA oligonucleotides listed in Supplementary Table S1 were purchased from Sangon Biotech. Stock solutions were prepared at 1 mM concentrations in H_2_O and stored at -25°C. The fluorophore 22R DNA (F-22R-T) was obtained by labeling 6-FAM (donor) and TAMRA (acceptor) at the 5′- and 3′-ends, respectively. F-22R-T was prepared in H_2_O to 0.1 mM and stored in the dark at –25°C. Samples in the experiments were prepared in buffers as specified in the figure captions. The GQ-folding was achieved by heating (95°C for 5 min) and gradually cooling to ambient temperature.

### Protein expression and purification

The truncated HMGB1 protein containing the A box (aa 9–78, termed HMGB1 in this paper) was expressed and purified as previously reported (29). The concentration of HMGB1 was determined through UV absorbance at 280 nm using the extinction coefficient ϵ = 9970 M^−1^cm^−1^. The FITC-labeled HMGB1 was prepared by incubation of FITC with HMGB1 for 8 h at 4°C in the dark. The precipitate was removed through centrifugation and the unreacted FITC molecules were removed through dialysis followed by ultrafiltration.

Five mutants of HMGB1, including single site mutations of R70A, Y71A and H31A, triple site mutation of K44A/K50A/K65A, and quadruple site mutation of K44A/K50A/K65A/R70A, were constructed. The primers used for constructing mutants were as listed in Supplementary Table S2. The plasmids were verified through sequencing, and the proteins were characterized by gel electrophoresis and ESI-MS.

### Nuclear magnetic resonance (NMR) spectroscopy

^1^H NMR and 2D ^1^H–^15^N HSQC spectra were collected on a Bruker Avance 600 MHz NMR spectrometer equipped with a TCI CryoProbe at 25°C. ^1^H-NMR spectra were recorded on 100 μM 22R in K_2_HPO_4_/KH_2_PO_4_ buffer (pH 7.4) containing 120 mM K^+^ at 25°C. ^1^H–^15^N HSQC spectra were collected on 150 μM ^15^N-labeled HMGB1 in the absence or the presence of different concentrations of GQ^KRAS^ at 25°C. Samples were all prepared in buffer containing 10% D_2_O, and the water signal was suppressed by the WATERGATE pulse sequence. The data were processed and analyzed using Sparky software.

### Circular dichroism (CD) spectroscopy

CD spectra were recorded on a Jasco J-810 CD spectrometer flashed with high purity nitrogen gas. Samples were placed in a 1.0 mm path length quartz cuvette. All spectra were recorded from 320 to 200 nm at a scan speed of 100 nm·min^−1^ with a data pitch of 1 nm. Spectra of buffer without proteins and oligonucleotides were also recorded for baseline corrections.

CD thermal analyses were carried out by recording CD spectra of 10 μM GQ^KRAS^ in the absence or presence of HMGB1 at different temperatures. The melting curves were processed using the ellipticity at 264 nm, which is one of the characteristic signals of parallel GQ and is not interfered with by the addition of HMGB1.

### Fluorescence titration

Fluorescence measurements were carried out on a Hitachi F 4600 fluorescence spectrophotometer. The excitation wavelength was set at 280 nm, and the emission fluorescence spectra were recorded from 290 to 450 nm. The fraction of GQ^KRAS^-bound HMGB1 (α) during the fluorescence titration of GQ^KRAS^ was calculated by the formula (1) according to literature (26).


(1) \begin{equation*}\alpha = \frac{{{I}_{335} - I_{335}^{free}}}{{I_{335}^{bound} - I_{335}^{free}}}\end{equation*}

where $I_{335}^{free}$ and $I_{335}^{bound}$ are the fluorescence intensities at 335 nm of 10 μM free and fully GQ^KRAS^-bound HMGB1, respectively. ${I}_{335}$ is the fluorescence intensity of HMGB1 at 335 nm during the titration at given concentrations of GQ^KRAS^.

### Förster resonance energy transfer (FRET) analysis

FRET experiments were carried out using 200 nM 22R labeled with 6-FAM and TAMRA at the 5′- and 3′-termini (F-22R-T), respectively. The fluorescence emission spectra were obtained by setting the excitation wavelength at 480 nm and detection from 490 to 700 nm.

The ratio of GQ-folding of the KRAS promoter DNA was assessed through FRET efficiency using the following formula (2) (30).


(2) \begin{equation*}{E}_{FRET} = \frac{{{I}_d}}{{{I}_d + {I}_a}}\end{equation*}

where *I_d_* is the fluorescence intensity at 520 nm of the donor (6-FAM) and *I_a_* is the fluorescence intensity at 590 nm of the acceptor (TAMRA).

### Phase separation prediction

Bioinformatic tools PrDOS and PLAAC were used to predict the phase separation tendency of protein HMGB1. Residues with a score >0.5 were regarded as intrinsically disordered regions (31,32).

### Turbidity assay

Turbidity assay was performed to analyze the extent of phase separation *in vitro*. GQ^KRAS^ and HMGB1 samples were prepared in 10 mM HEPES buffer (pH 7.4) containing K^+^ and 10% PEG_8K_. The turbidity of the samples was first assessed through the absorbance at 400 nm by a SpectraMax^®^ Quickdrop™ UV–vis spectrophotometer with a light path of 0.5 mm.

### Droplet fluidity assay

Phase separation samples were prepared in 10 mM HEPES buffer (pH 7.4) containing 10% PEG_8K_ with the indicated concentrations of K^+^. The droplets were observed on an inverted fluorescence microscope (Olympus IX73) equipped with a 100× oil immersion objective lens. The formation and fusion droplets were observed under the DIC mode. Fluorescent images of droplets were collected by excitation of corresponding fluorophores (EtBr, ThT or FITC) labeled to GQ^KRAS^ or HMGB1. Liquid droplet fusion assay was carried out on droplets formed by 50 μM GQ^KRAS^ with 100 μM HMGB1 in the presence of 10% PEG_8K_, and the images were recorded on droplets in a time-lapse manner under DIC mode.

Fluorescence recovery after photobleaching (FRAP) assays were performed on GQ^KRAS^ (50 μM) and FITC-labeled HMGB1 (100 μM) in 10 mM HEPES buffer (pH 7.4) containing 10% PEG_8K_ and 20 mM K^+^ ions. The sample was dropped on a glass slide with a coverslip and observed under a laser confocal microscope. After bleaching a droplet with a 488 nm laser, the fluorescence intensity of the bleaching area was monitored. The fluorescence intensity of an unbleached region was monitored as a reference. Images were recorded with intervals of 5 s to monitor the fluorescence change in the bleaching areas, and the fluorescence recovery profiles were processed using Origin 2023.

## Results

### Interaction of HMGB1 with GQ^KRAS^

The formation of GQ structure of the 22R sequence (shown in Supplementary Figure S1A) used in this work has been verified by using NMR and circular dichroism (CD) spectroscopies. The ^1^H NMR spectrum (Supplementary Figure S1B) showed a group of peaks in the range of 10–12 ppm, indicating the typical imino protons involved in Hoogsteen base pairings in GQ, which is consistent with the literature results (33,34). CD spectra showed the signature bands of parallel GQ, positive at 264 nm and negative at approximately 243 nm (Supplementary Figure S1C). These results confirmed the formation of the GQ topology of 22R under the experimental conditions.

It has been reported that HMGB1 can interact with KRAS GQs (26). In this work, a truncated HMGB1 containing the A box with a well-folded structure (aa 9–78, termed HMGB1 hereafter) was used, and its interaction with GQ^KRAS^ was verified by different approaches. All these measurements were performed in the presence of K^+^ ions to allow GQ folding of 22R. Electrophoretic mobility shift assay (EMSA) showed that incubation of HMGB1 and GQ^KRAS^ formed a complex band with lower mobility (Supplementary Figure S2A). Size exclusion chromatography (SEC) clearly indicated the generation of the GQ^KRAS^/HMGB1 complex with a smaller retention volume (Supplementary Figure S2B). Fluorescence measurements showed that the titration of GQ^KRAS^ quenched the fluorescence of HMGB1 at 335 nm in a concentration-dependent manner (Supplementary Figure S2C and D). Fitting the titration curve gave an equilibrium dissociation constant (K_d_) of 4.08 μM. Moreover, isothermal titration calorimetry (ITC) was performed to directly measure the binding enthalpies and thermodynamic parameters of interactions (Supplementary Figure S2E and S2F). The obtained *K*_d_ of 7.04 μM is in good agreement with the result of fluorescence titration. The thermodynamic parameters showed that the interaction of GQ^KRAS^ and HMGB1 gave a highly negative enthalpy (Δ*H* = –6.457 ± 0.537 kcal/mol) and a small positive entropy (*T*Δ*S* = 0.572 kcal/mol). These values (|Δ*H*| > |*T*Δ*S*|) indicate that the interaction is mainly an enthalpy-driven process, probably derived from electrostatic effects (35,36).

### Co-condensation of GQ^KRAS^ with HMGB1

**Figure 1.**
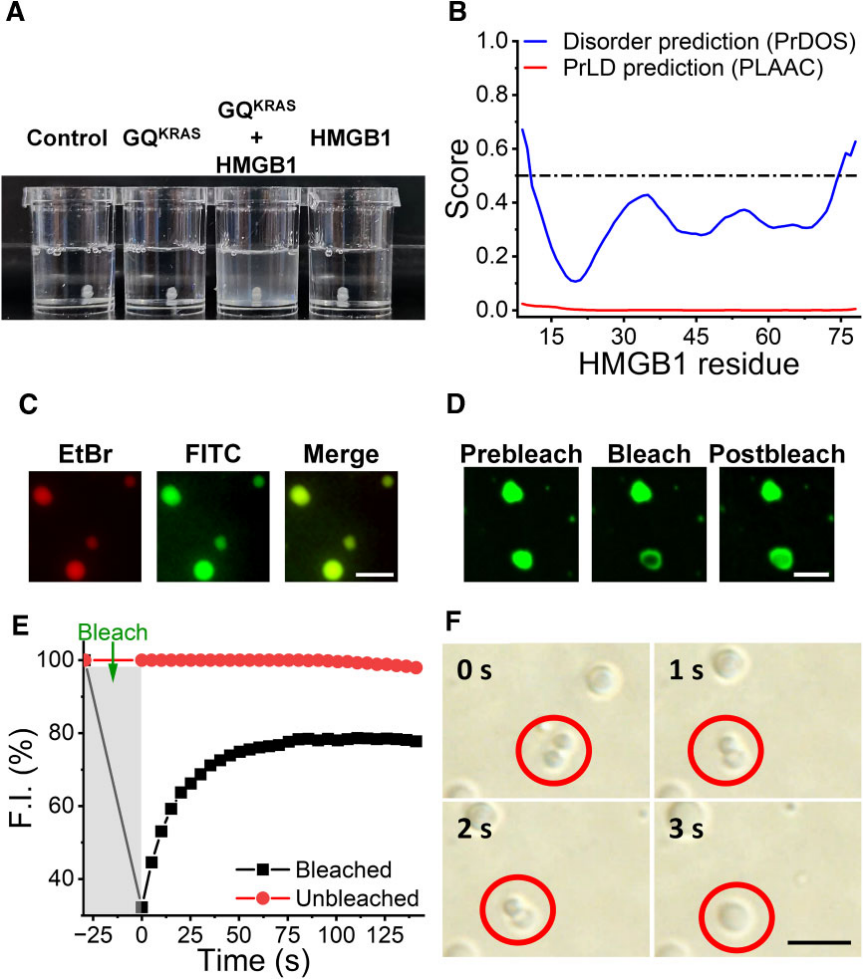
Co-condensation of GQ^KRAS^ and HMGB1. (**A**) Turbidity analysis of GQ^KRAS^ and HMGB1. The experiment was performed on 25 μM GQ^KRAS^ and 50 μM HMGB1 in the presence of 10% PEG_8K_ in 10 mM HEPES (pH 7.4) containing 20 mM K^+^. Buffer with PEG_8K_ was used as a control. (**B**) Phase separation prediction of HMGB1. Prediction of intrinsically disordered regions of HMGB1 using PrDOS(32) (blue) and assessment of prion-likeness by PLAAC(31) (red). Values of PrDOS and PLAAC above 0.5 signify residues in disordered region and high prion tendencies, respectively. (**C**) Fluorescence microphotographs of liquid droplets formed by 50 μM GQ^KRAS^ and 100 μM HMGB1. GQ^KRAS^ was stained with EtBr (red), and HMGB1 was labeled with FITC (green). Scale bar: 10 μm. (**D**) Fluorescence recovery after photobleaching experiments in GQ^KRAS^/HMGB1 droplets. The droplets were formed by 50 μM GQ^KRAS^ and 100 μM HMGB1 in the presence of 10% PEG_8K_ in 10 mM HEPES (pH 7.4) containing 20 mM K^+^. Scale bar: 5 μm. (**E**) Fluorescence recovery profile corresponding to Figure 1D. (**F**) Droplet fusion observed on time-lapse images. The droplets were formed by 50 μM GQ^KRAS^ with 100 μM HMGB1. Scale bar: 10 μm.

It has been reported that GQ-folding of DNA could promote LLPS of several proteins enriched in nucleus (27). Hence, we hypothesized that the interaction of GQ^KRAS^ and HMGB1 could influence the phase behavior of the two types of biomolecules. First, we verified whether GQ^KRAS^ or HMGB1 alone could undergo LLPS. Turbidity analysis showed that neither GQ^KRAS^ (25 μM) nor HMGB1 (50 μM) formed LLPS in the presence of a crowding agent (10% PEG_8K_) (Figure 1A). Intriguingly, the mixture of GQ^KRAS^ and HMGB1 clearly turned turbid under the same conditions, suggesting the higher LLPS tendency of the mixture, while the presence of PEG_8K_ did not alter the folding of either GQ^KRAS^ or HMGB1 (Supplementary Figure S3A). By comparison, no phase separation occurred even at high concentrations of either GQ^KRAS^ (200 μM, Supplementary Figure S3B) or HMGB1 (400 μM, Supplementary Figure S3C). This result clearly indicated that the interaction of GQ^KRAS^ with HMGB1 mutually promotes the LLPS process of the two biomacromolecules.

The intrinsic disordered regions (IDRs) and prion-like domains (PrLDs), which are the key characteristics predicting the ability for protein LLPS, were analyzed using PrDOS and PLAAC methods, respectively (31,32,37). HMGB1 possesses only very short IDRs at two termini, and its PLAAC score is far below 0.5 (Figure 1B). These values imply that HMGB1 features a well-folded structure and has a very low tendency to form LLPS, which is consistent with the turbidity analysis.

The formation of LLPS and coexistence of GQ^KRAS^ and HMGB1 in the condensates were further verified using fluorescence microscopy. To distinguish the two components, GQ^KRAS^ was stained with ethidium bromide (EtBr), which shows red fluorescence under irradiation at 543 nm. Meanwhile, HMGB1 was labeled with fluorescein isothiocyanate (FITC), which shows green fluorescence under irradiation at 488 nm. Fluorescence microscopy measurements clearly showed that both GQ^KRAS^ and HMGB1 can be observed in the same droplets (Figure 1C), confirming the co-condensation of GQ^KRAS^ and HMGB1.

The liquid-like property of droplets was characterized by the fluorescence recovery after photobleaching (FRAP) method. The fluorescence of the photo-bleached region in droplets can be gradually recovered to approximately 80% in one minute (Figure 1D and E, Supplementary Movie S1), referenced to unbleached droplets. Additionally, time-lapse microscopy clearly showed the rapid fusion of small droplets into large droplets. (Figure 1F, Supplementary Movie S2). These results indicate that the droplets formed by GQ^KRAS^ and HMGB1 were highly dynamic in a liquid state, in accordance with the characteristics of LLPS.

### Quadruplex folding is required for DNA to co-condensate with HMGB1

**Figure 2.**
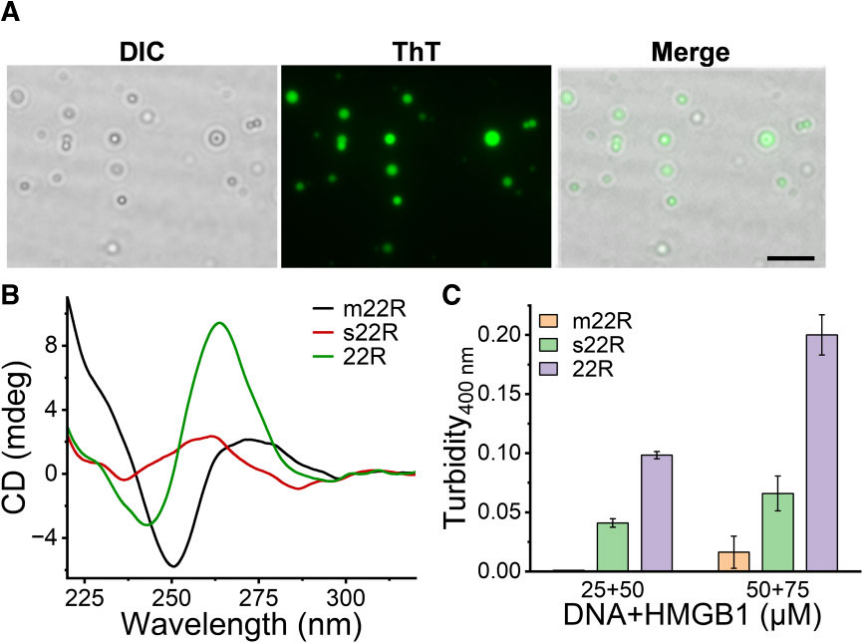
Analysis of the GQ-folding of 22R in the co-condensates of GQ^KRAS^/HMGB1. (**A**) Microscopic imaging of GQ^KRAS^/HMGB1 co-condensates. Images were recorded with differential interference contrast (DIC) or fluorescence imaging. The GQ^KRAS^/HMGB1 droplets were formed by 50 μM GQ^KRAS^ with 100 μM HMGB1 in the presence of 50 μM ThT. Scale bar: 10 μm. (**B**) CD spectra analysis of different DNA sequences (10 μM) in 10 mM HEPES buffer (pH 7.4) containing 20 mM K^+^. (**C**) Turbidity analysis of difference DNA sequences with HMGB1 in 10 mM HEPES buffer (pH 7.4) containing 20 mM K^+^ and 10% PEG_8K_.

Next, we verified whether the co-condensation of GQ^KRAS^ and HMGB1 relies on the GQ topology of 22R DNA. Incubation of the GQ^KRAS^/HMGB1 droplets with thioflavin T (ThT, a GQ fluorogenic probe) clearly showed the fluorescence of ThT inside the droplets (Figure 2A). Turbidity analysis indicated that ThT did not affect the LLPS of GQ^KRAS^/HMGB1 (Supplementary Figure S3D and E). This result suggests that the 22R DNA retained GQ-folding in the co-condensates with HMGB1.

To confirm the role of the GQ structure of 22R in the co-condensation with HMGB1, the assay was performed by alteration GQ folding in 22R sequence through G-to-A mutation of 22R (termed m22R, Supplementary Table S1), or using a scrambled sequence of 22R (termed as s22R, Supplementary Table S1). CD spectra confirmed that the two sequences (m22R and s22R) could not form GQ folding (Figure 2B). Turbidity analysis indicated that interferences with the GQ-folding significantly reduced the co-condensation with HMGB1 (Figure 2C). This result confirmed that the GQ structure is required for the co-condensation of 22R with HMGB1.

### HMGB1 promotes the co-condensation by facilitating GQ folding of the DNA

**Figure 3.**
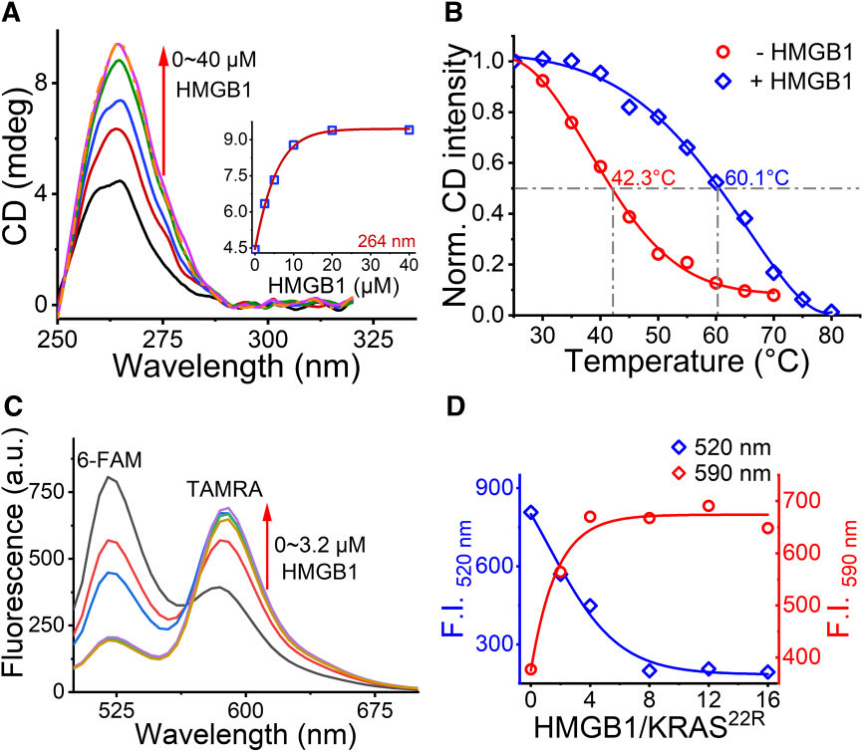
HMGB1 promotes GQ-folding of 22R. (**A**) CD spectra of 10 μM GQ^KRAS^ with different concentrations of HMGB1 (0 – 40 μM) in 10 mM HEPES (pH 7.4) containing 5 mM K^+^. The inset shows ellipticity of GQ^KRAS^ at 264 nm as a function of HMGB1 concentration. (**B**) CD thermal melting curves of 10 μM GQ^KRAS^ in the absence (red) or presence (blue) of 30 μM HMGB1 in 20 mM K_2_HPO_4_/KH_2_PO_4_ (pH 7.4) with 70 mM KCl. The CD intensity at 264 nm of 10 μM GQ^KRAS^ at 25°C was used as a reference. (**C**) Fluorescence spectra of 200 nM F-22R-T after incubation with different concentrations of HMGB1 with excitation at 480 nm. (**D**) Alteration of the fluorescence intensity of 6-FAM at 520 nm (blue) and TAMRA (red) at 590 nm in (C).

As the quadruplex structure of 22R is required for the co-condensation with HMGB1, we hypothesized that HMGB1 promotes the LLPS of 22R by facilitating its quadruplex folding. To verify this hypothesis, we analyzed the HMGB1-induced structural alteration of the 22R sequence. At a low K^+^ concentration (5 mM), 22R can only partially fold in the GQ structure. CD spectra showed that the characteristic peak of parallel GQ at 264 nm increased with the addition of HMGB1 (Figure 3A), indicating that HMGB1 promotes the quadruplex folding of 22R. Furthermore, the temperature-dependent CD experiment demonstrated that the presence of HMGB1 increased the melting temperature (*T*_m_, 50% disruption of GQ structure) of GQ^KRAS^ from 42.3°C to 60.1°C (Figure 3B, Supplementary Figure S4A and S4B). This result confirmed that HMGB1 significantly facilitated and stabilized the quadruplex folding of 22R.

Fluorescence resonance energy transfer (FRET) has also been applied to analyze the conformational change of 22R upon HMGB1 interaction. Two fluorophores, 6-FAM (donor) and TAMRA (acceptor), were labeled at the 5′- and 3′- termini of 22R (termed F-22R-T), respectively. Hence, the conformation of 22R can be detected by FRET since GQ folding reduces the distance between the two termini. The FRET of F-22R-T can be observed via emission of TAMRA at 590 nm with the excitation of 6-FAM at 480 nm. Adding HMGB1 clearly enhanced the fluorescence of TAMRA (590 nm), accompanied by the decreased fluorescence of 6-FAM (520 nm) (Figure 3C and D), showing the enhanced FRET efficiency of F-22R-T by HMGB1 in a concentration-dependent manner. This result further supports the conclusion that HMGB1 promoted the GQ folding of 22R.

### Properties of the GQ^KRAS^/HMGB1 co-condensation system

**Figure 4.**
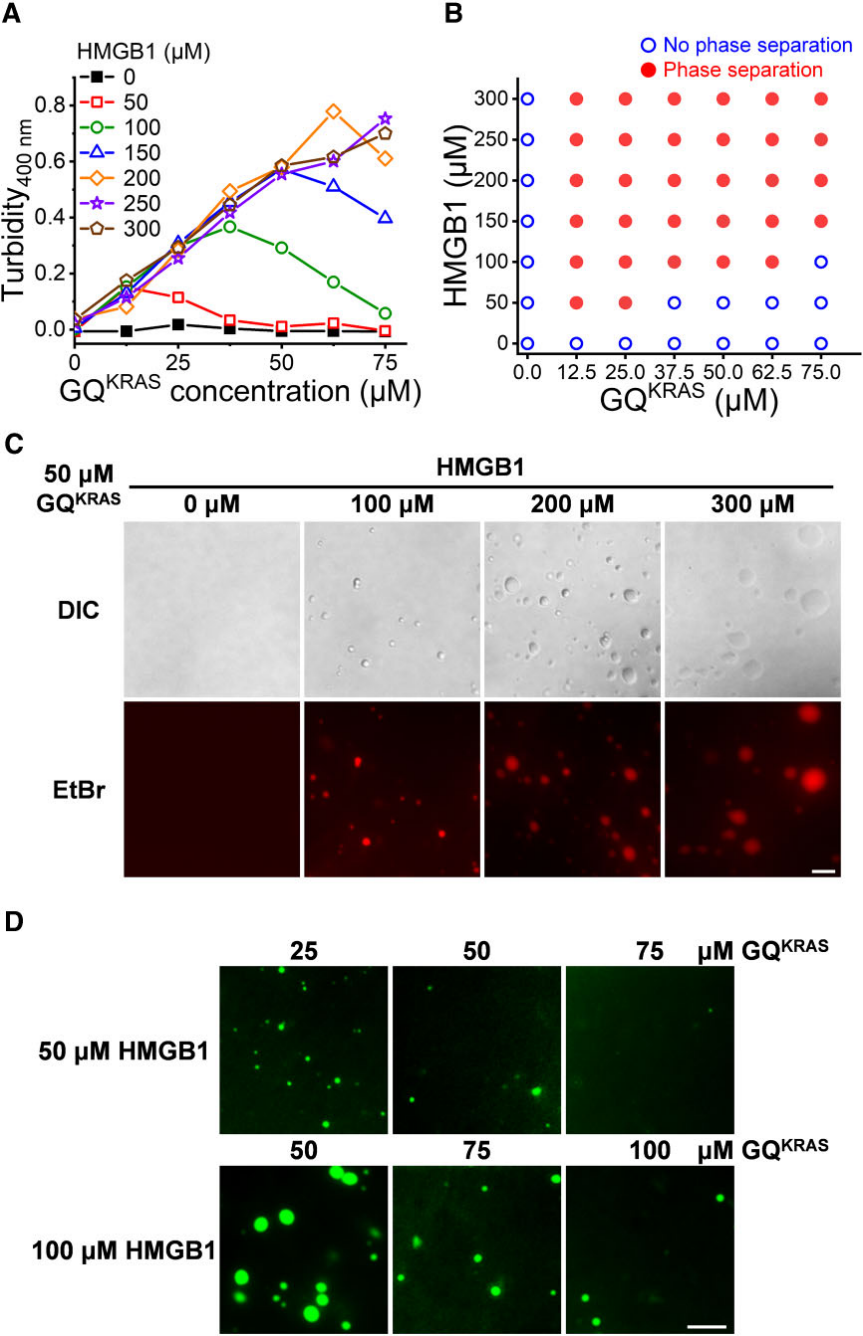
(**A**) Turbidity of GQ^KRAS^/HMGB1 condensates formed in 10 mM HEPES buffer (pH 7.4) containing 20 mM KCl and 10% PEG_8K_. (**B**) Phase diagrams of GQ^KRAS^ and HMGB1 under 20 mM K^+^. Blue circles indicate no phase separation, while red dots indicate phase separation. (**C**) Microscopic images of the droplets formed by 50 μM GQ^KRAS^ in the presence of increasing concentrations of HMGB1 as indicated. The samples above were prepared in 10 mM HEPES buffer (pH 7.4) containing 20 mM KCl and 10% PEG_8K_. Scale bar: 10 μm. (**D**) Images of the droplets formed by GQ^KRAS^ with HMGB1 (labeled with FITC) in the presence of increasing concentrations of GQ^KRAS^ as indicated. The samples above were prepared in 10 mM HEPES buffer (pH 7.4) containing 20 mM KCl and 10% PEG_8K_. Scale bar: 10 μm.

The co-condensation of GQ^KRAS^ and HMGB1 has been systematically analyzed at different concentrations. Turbidity measurement clearly showed the correlation of LLPS with the concentrations of GQ^KRAS^ and HMGB1 (Figure 4A and B). Interestingly, HMGB1 and GQ^KRAS^ demonstrated different effects on phase separation. While the turbidity continuously increased with the concentration of HMGB1, GQ^KRAS^ increased the phase separation depending on the ratio of HMGB1 to GQ^KRAS^. At a certain concentration of HMGB1, increasing the concentration of GQ^KRAS^ enhanced condensation at the early stage, and the turbidity reached a maximum at a critical ratio ([HMGB1]/[GQ^KRAS^] = 3–4) (Figure 4A and B). Further addition of GQ^KRAS^, which lowered the ratio of [HMGB1]/[GQ^KRAS^], reduced the turbidity; and the LLPS of HMGB1 and GQ^KRAS^ completely disappeared at the 1:1 ratio. This result suggests that, although HMGB1 interacts with GQ^KRAS^ and forms protein/DNA complex, additional HMGB1 is requisite for the phase separation of GQ^KRAS^/HMGB1 (Supplementary Figure S5). Moreover, fluorescence imaging also confirmed the different concentration dependences of LLPS on HMGB1 and GQ^KRAS^; the number and size of droplets continuously increased with the concentration of HMGB1 (Figure 4C and Supplementary Figure S6A), confirming the hypothesis that HMGB1 promotes the condensation by facilitating and stabilizing GQ^KRAS^, as mentioned above. Nevertheless, it can be clearly observed that in the well phase-separated system, in which excessive HMGB1 were present, addition of GQ^KRAS^ caused dwindling and diminishing droplets (Figure 4D and Supplementary Figure S6B). This result confirmed that excessive HMGB1 is required for the co-condensation of GQ^KRAS^.

### Driving forces of the co-condensation of GQ^KRAS^ and HMGB1

**Figure 5.**
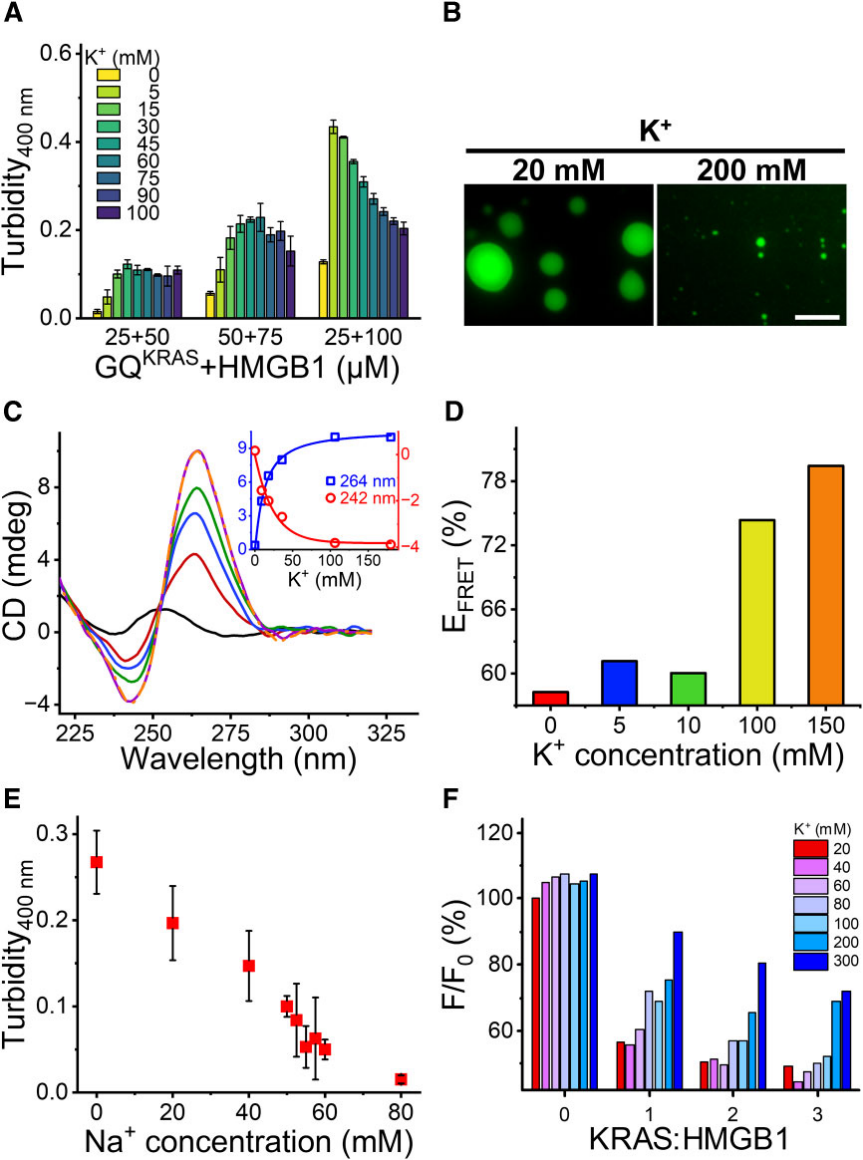
Effect of K^+^ ions on the LLPS of GQ^KRAS^/HMGB1. (**A**) Turbidity of GQ^KRAS^/HMGB1 with different concentrations of K^+^ ions. The measurement was performed in 10 mM HEPES buffer (pH 7.4) containing 10% PEG_8K_. (**B**) Images of the droplets formed by 50 μM GQ^KRAS^ with 100 μM HMGB1. The samples above were prepared in 10 mM HEPES buffer (pH 7.4) containing 20 mM or 200 mM K^+^ as indicated with 10% PEG_8K_. Scale bar: 10 μm. (**C**) CD spectra of 10 μM 22R in H_2_O (black) or in K_2_HPO_4_/KH_2_PO_4_ buffer (pH 7.4) containing 0–180 mM K^+^. The inset plot shows the ellipticity of GQ^KRAS^ monitored at 264 nm (blue squares) and 242 nm (red circles) as a function of K^+^ concentration. (**D**) FRET efficiency of F-22R-T (200 nM) in 10 mM HEPES buffer (pH 7.4) containing 0–150 mM K^+^ as indicated. (**E**) Turbidity analysis of the co-condensation of GQ^KRAS^/HMGB1 as a function of Na^+^ concentration. Samples were prepared with 25 μM GQ^KRAS^ and 75 μM HMGB1 in 10 mM HEPES buffer (pH 7.4) containing 30 mM K^+^ and 10% PEG_8K_. (**F**) Alteration of fluorescence intensity (at 350 nm) of HMGB1 in the absence or presence of GQ^KRAS^ at different concentrations of K^+^ ions. *F*_0_ is the initial fluorescence intensity of 10 μM HMGB1, and *F* is the fluorescence intensity at the given concentration of GQ^KRAS^ and K^+^ ions.

The different effects of GQ^KRAS^ on the co-condensation of GQ^KRAS^/HMGB1 encouraged further exploration of the driving force of the co-condensation. First, we analyzed the effect of K^+^ concentration on the co-condensation system, as K^+^ ions are generally required to stabilize the GQ-folding of G-rich sequences (38). Turbidity analysis indicated that the phase separation of GQ^KRAS^ and HMGB1 was promoted by K^+^ ions at a low concentration range. The turbidity reached a maximum at a critical K^+^ concentration, for example, 30 mM K^+^ ions for 50 μM GQ^KRAS^ and 75 μM HMGB1. Unexpectedly, the turbidity decreased with further addition of K^+^ ions (Figure 5A and B), suggesting that K^+^ ions at high concentrations could reduce the LLPS of GQ^KRAS^/HMGB1. This effect was observed with different concentrations of GQ^KRAS^ and HMGB1, although the optimal concentration of K^+^ ions varied with DNA concentration.

To clarify the different effects of K^+^ ions on the LLPS of GQ^KRAS^/HMGB1, the folding of the 22R sequence at different K^+^ concentrations was verified. CD spectroscopy indicated that the elliptical intensity of the characteristic signal of parallel GQ (264 nm) continuously increased with the addition of K^+^ ions (Figure 5C). In addition, ^1^H-NMR spectra demonstrated that the titration of K^+^ ions induced the formation of GQ-folding, indicated by the characteristic imino-proton signals in the chemical shift region of 10.5–12 ppm (Supplementary Figure S7). FRET efficiency was also enhanced at high K^+^ concentrations (Figure 5D). The peak alterations of aromatic protons (7.0–8.5 ppm) shows the conversion of DNA conformation from single strand to quadruplex with the increase of K^+^ concentrations, and the GQ-folding formed dominantly in the presence of 36 mM or higher concentration of K^+^ ions. No disruption of GQ-folding was observed at high K^+^ concentration. This result confirmed that K^+^ stabilized GQ^KRAS^ even at high K^+^ concentrations. Hence, the decreased LLPS of GQ^KRAS^/HMGB1 at high K^+^ concentrations is not caused by structural alteration of GQ^KRAS^.

We hypothesized that extra K^+^ ions could partially disrupt the electrostatic interaction between GQ^KRAS^/HMGB1 complex and additional HMGB1 protein, particularly the weak multivalent interactions leading to the formation of LLPS. To verify this hypothesis, we tested the effect of cationic ions on the formation of GQ^KRAS^/HMGB1 droplets in the presence of additional Na^+^ ions. Turbidity analysis showed that, at an optimal K^+^ concentration, adding Na^+^ ions clearly decreased the turbidity in a Na^+^ concentration-dependent manner (Figure 5E), similar to the addition of extra K^+^ ions. This result suggests that high concentrations of cationic ions (K^+^ or Na^+^) can inhibit the co-condensation of GQ^KRAS^/HMGB1, probably by interfering with their electrostatic interactions.

The attenuation of the GQ^KRAS^/HMGB1 interaction by high concentration of K^+^ ions was further confirmed by fluorescence spectroscopy. The result showed that, at a certain K^+^ concentration, the intrinsic fluorescence of HMGB1 was clearly reduced by GQ^KRAS^ in a concentration-dependent manner (Supplementary Figure S2C), indicating the interaction of the two biomolecules. Nevertheless, increasing the K^+^ concentration partially recovered the fluorescence of HMGB1 quenched by GQ^KRAS^, while the fluorescence of HMGB1 alone was barely affected by K^+^ ions (Figure 5F and Supplementary Figure S8). In addition, we also analyzed the effect of K^+^ ions on the GQ^KRAS^/HMGB1 interaction by measuring the binding affinity at different K^+^ concentrations. Fitting the fluorescence titration gave the dissociation constants of 3.24 × 10^−6^ M and 4.24 × 10^−6^ M in 20 and 200 mM K^+^ ions, respectively (Supplementary Figure S9). The values indicated that high concentrations of K^+^ ions slightly attenuated the interaction between HMGB1 and GQ^KRAS^, which could only partially affect their co-condensation. This result supports the hypothesis that the perturbation of co-condensation of GQ^KRAS^/HMGB1 by high concentration of K^+^ ions mainly results from the interference of electrostatic interactions between GQ^KRAS^/HMGB1 complexes and additional HMGB1 protein.

**Figure 6.**
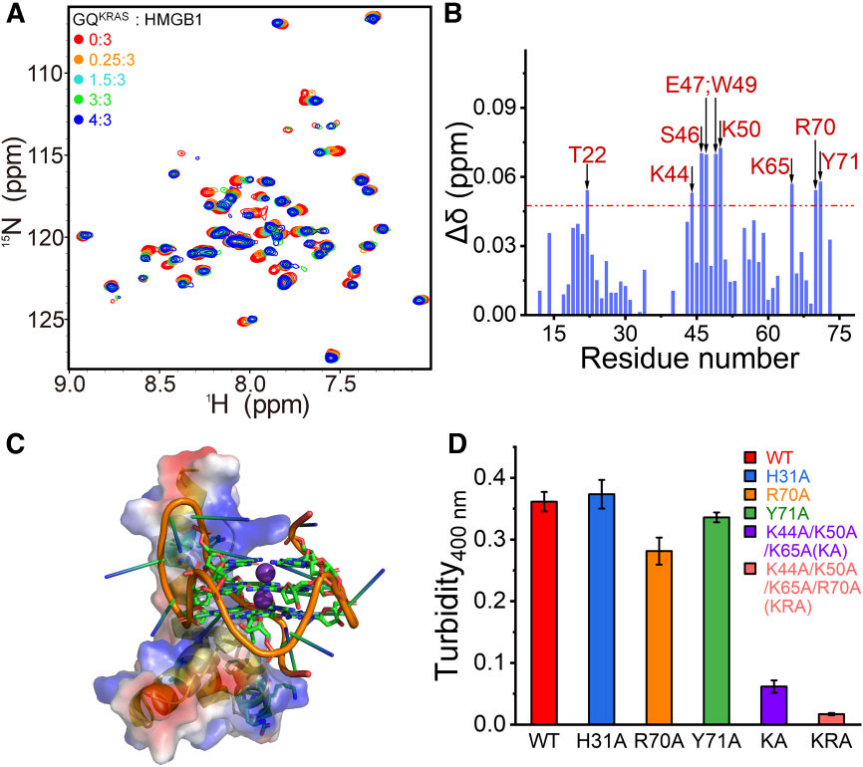
(**A**) Overlay of ^1^H-^15^N HSQC spectra of 150 μM HMGB1 before (red) and after reaction with different concentrations of GQ^KRAS^ as indicated. (**B**) Chemical shift perturbation (CSP) plot of HMGB1 in the presence of 150 μM GQ^KRAS^ as a function of residue number. CSP was calculated as $\Delta \delta = \sqrt {[{{( {\Delta {\delta }_H} )}}^2 + {{( {\Delta {\delta }_N/5} )}}^2]/2}$. The dashed line indicates the threshold of the chemical shift changes calculated based on the average chemical shift across all residues plus the standard deviation (44). (**C**) Molecular docking of the GQ^KRAS^/HMGB1 complex using online HDOCK SERVER (http://hdock.phys.hust.edu.cn/). The DNA is present in stick mode and HMGB1 was shown in space filling mode, and the color denotes negatively charged surface (red) or positively charged surface (blue). The image was processed with PyMOL software. (**D**) Mutagenesis analysis of the effect of key residues of HMGB1 on the co-condensation with GQ^KRAS^. Turbidity was measured on GQ^KRAS^ (50 μM) and HMGB1 mutants (100 μM) in 10 mM HEPES buffer (pH 7.4) containing 20 mM K^+^ and 10% PEG_8K_.

Since the protein structure is an important factor for the formation of condensates, the folding of HMGB1 in the interaction with GQ^KRAS^ has been verified using 2D NMR spectroscopy on ^15^N-labeled HMGB1. Upon titration of GQ^KRAS^, the HMGB1 peaks exhibited only slight shifts, while the pattern of the whole spectra remained nearly unchanged (Figure 6A and B), suggesting that the folding of HMGB1 was negligibly affected in the interaction with GQ^KRAS^. Furthermore, molecular docking results showed that the residues exhibiting relatively large chemical shift changes in the NMR titration are mainly located on the interaction interface in the GQ^KRAS^/HMGB1 complex (Figure 6C), confirming the significance of structural recognition on their interaction and co-condensation.

To further explore the effect of GQ^KRAS^/HMGB1 interaction on the co-condensation, we analyzed the interaction interface of the complex and constructed five mutants of HMGB1, including single site mutations of R70A, Y71A and H31A, triple site mutation of K44A/K50A/K65A, and quadruple site mutation of K44A/K50A/K65A/R70A. Residues K44, K50, K65, R70 and Y71 are on the interaction interface of HMGB1 protein and their NMR signals were perturbed in the GQ^KRAS^ titration (Figure 6), while H31 is not involved in the interaction. After purification, the identities of mutants were verified by gel electrophoresis and ESI-MS (Supplementary Figure S10). The co-condensation of mutants with GQ^KRAS^ was explored through turbidity analysis (Figure 6D). In comparison to the wild type HMGB1 (WT), H31A mutation did not alter the co-condensation with GQ^KRAS^, confirming that the phase separation was not significantly affected by residues that are not involved in the interaction with GQ^KRAS^. Single mutation of positive-charged residue (R70A) demonstrated more influence than the mutation of aromatic residue (Y71A) on the interaction interface. As expected, the triple and quadruple mutations of positive-charged residues (K44A/K50A/K65A and K44A/K50A/K65A/R70A) almost fully disrupted the phase separation. This result confirmed that the interaction between GQ^KRAS^ and HMGB1 plays a key role in the co-condensation of the GQ^KRAS^/HMGB1 system.

To further verify the specificity of GQ^KRAS^ in the co-condensation with HMGB1, several other DNA sequences (termed as LTRIII, 2G and h-Tel, the sequences given in Supplementary Table S1) that can form quadruplex structures were explored. Turbidity analysis indicated that the incubation of 22R with HMGB1 clearly formed phase separation (Supplementary Figure S11). Under the same experimental conditions, much lower degree of phase separation was observed with LTRIII sequence, while nearly no phase separation formed on the other two sequences. The result confirmed the high selectivity of GQ^KRAS^ in the co-condensation with HMGB1.

To further verify the driving force of LLPS of GQ^KRAS^ and HMGB1, the condensates were treated with 1,6-hexanediol, a specific LLPS inhibitor disrupting hydrophobic interactions. The turbidity result showed that LLPS was hardly affected by 1,6-hexanediol at concentrations as high as 20% (v/v) (Supplementary Figure S12). This result indicates that hydrophobic interaction is not the main driving force of co-condensation of GQ^KRAS^ and HMGB1. These results suggest that electrostatic interactions and GQ formation play prominent roles in the co-condensation of negatively charged GQ^KRAS^ and positively charged HMGB1. It is well-known that hydrophobicity-mediated phase separation is a common feature in protein LLPS; nevertheless, electrostatic interactions and other hydrophilic interactions were found playing major roles in some phase separation systems, such as tau protein, tau/prion complex and VRN1/DNA complex (39–41). Here we found electrostatic interaction is the dominant driving force in the co-condensation of G-quadruplex and its binding protein.

## Discussion

**Figure 7.**
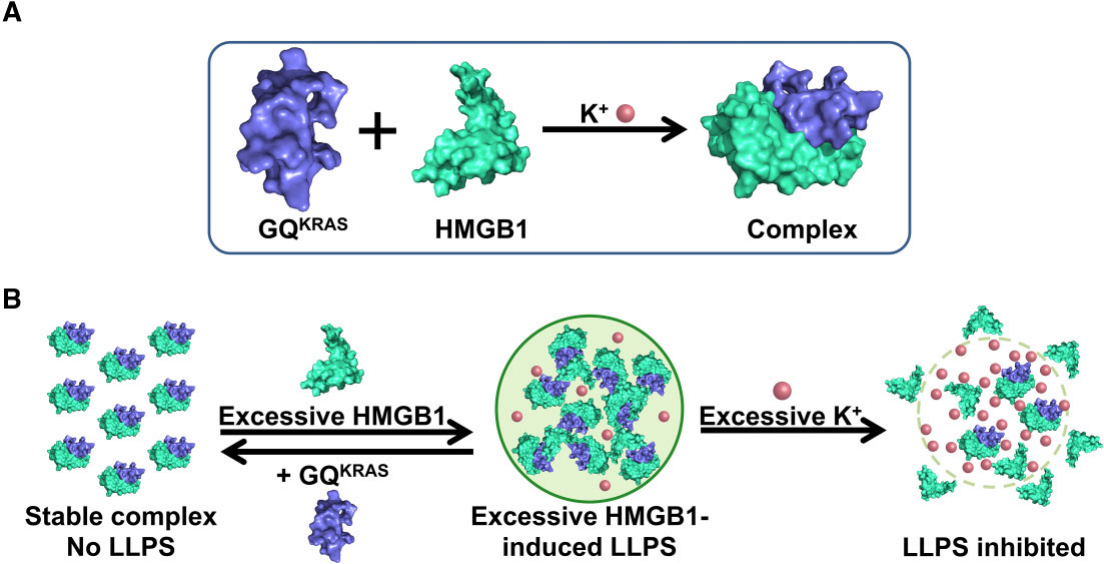
Schematic illustration of formation and disruption of the condensation of GQ^KRAS^/HMGB1. (**A**) Interaction of GQ^KRAS^ and HMGB1. The complex structure was obtained by molecular docking using online HDOCK SERVER (http://hdock.phys.hust.edu.cn/) and processed with PyMOL software. (**B**) Excessive HMGB1-induced co-condensation of GQ^KRAS^ and HMGB1, which is disrupted by excessive K^+^.

Quadruplex folding of G-rich sequences is involved in many important physiological and pathological processes, while the formation of LLPS could modulate the functions of GQs. On the other hand, the GQ-folding is typically regulated by their binding proteins (42). In the present study, we found that the HMGB1 protein facilitates the GQ-folding of the KRAS promoter sequence. Although neither GQ^KRAS^ nor HMGB1 tends to form LLPS, their interaction promotes the co-condensation of two biomolecules. Furthermore, the ratio of HMGB1 to GQ^KRAS^ is crucial for their co-condensation, in agreement with the electrostatic interaction. NMR and ITC titrations confirmed that HMGB1 can interact with GQ^KRAS^ and form stable complex, which is supported by structural docking of the GQ^KRAS^/HMGB1 complex (Figure 6C). Nevertheless, the formation of stable complex is not sufficient to form LLPS of two biomacromolecules; additional HMGB1 is required for their co-condensation. It can be speculated that, in addition to the specific interaction between GQ^KRAS^ and HMGB1 that forms GQ^KRAS^/HMGB1 complex, the additional positively charged HMGB1 can interact with multiple GQ^KRAS^ molecules in GQ^KRAS^/HMGB1 complex through non-specific interactions resulting in multivalent weak interactions between GQ^KRAS^/HMGB1 units (Figure 7). In this circumstance, HMGB1 plays two functional roles in the co-condensation with GQ^KRAS^. On one hand, HMGB1 promotes and stabilizes the GQ-folding of GQ^KRAS^ by formation of GQ^KRAS^/HMGB1 complex; which is required for their co-condensation. On the other hand, the non-specific interaction between GQ^KRAS^/HMGB1 complex and additional HMGB1 enhances the phase separation. CD spectroscopy indicated that 22R DNA nearly completely folded into GQ structure already in the presence of equimolar of HMGB1 (Figure 3A). However, only very small degree of phase separation was formed in equimolar ratio, for example at 50 μM HMGB1 and 50 μM GQ^KRAS^, while additional HMGB1 significantly enhanced the phase separation (Figure 4A). Therefore, HMGB1 plays two functional roles in the co-condensation depending on the HMGB1/GQ^KRAS^ ratio. At equivalent or lower molar ratios, HMGB1 forms GQ^KRAS^/HMGB1 complex and stabilizes the GQ-folding of DNA; in this circumstance, very low level LLPS can be formed. LLPS is mainly caused by excessive HMGB1, which interacts with GQ^KRAS^/HMGB1 complex and induces co-condensation through non-specific multivalent interactions. Given that quadruplexes of DNA/RNA and their protein interactions exist universally in cells, the results in this work suggest that proteins could regulate the function of GQs by promoting their LLPS.

In addition to the ratio of HMGB1 to GQ^KRAS^, the concentration of K^+^ ions is also critical for the co-condensation of two biomolecules. The co-condensation GQ^KRAS^/HMGB1 is enhanced by K^+^ ions at relatively low concentration ranges, but suppressed by excessive K^+^ ions. Therefore, K^+^ concentration modulates the degree of the co-condensation, and the highest phase separation occurs at optimal K^+^ concentrations. The optimal K^+^ concentrations in cell could be different from that in solution, as the K^+^ ions in cells have different accessibility due to the restraint of various interactions. In addition, intracellular environment contains a large variety of biomolecules and salts that may influence the condition of phase separation. As various cellular compartments possess different micro-environments, and they can also change during cell viability, the finding of the effect of K^+^ ions suggests that the co-condensation of GQ and its binding-protein could be regulated in cells in response to environmental change during cellular processes.

Transient and multivalent weak interactions among macro-biomolecules, including electrostatic, hydrophobic, electron-donating and π–π interactions, are the key driving forces in the formation of liquid-like droplets. Molecular structures and dynamics play important roles in these interactions; it is generally believed that flexible sequences, such as IDRs in proteins, are favorable regions for protein condensation. In addition, protein droplets prefer to recruit flexible ss-DNA rather than rigid ds-DNA (43). It has been reported that IDRs of unfolded histone H1 contribute to its co-condensation with GQ DNA (27). In this work, however, two types of highly rigid molecules, HMGB1 protein and GQ^KRAS^, were found to form co-condensates in a mutual promotion manner. Mechanistic investigations revealed that electrostatic interactions play a pivotal role in their co-condensation, whereas hydrophobic interactions are negligible for these highly hydrophilic molecules. The ITC measurement confirms that the GQ^KRAS^/HMGB1 interaction is an enthalpy-driven process, consistent with electrostatic interactions (35,36). On the other hand, incubation of HMGB1 with ssDNA that could not form GQ (termed Random, see Supplementary Table S1) did not cause LLPS (Supplementary Figure S13). This result indicates that the electrostatic interaction is not the sole driving force of the co-condensation of GQ^KRAS^/HMGB1, while structural recognition also plays important roles in the mutual promotion of the co-condensation of two macro-biomolecules.

## Conclusion

In summary, we have demonstrated that the well-folded protein HMGB1 and KRAS G-quadruplex (GQ^KRAS^) can co-condensate in a mutual promotion manner, although either of them can hardly undergo phase separation. HMGB1 can interact with GQ^KRAS^ through an enthalpy-driven process, and the interaction facilitates and stabilizes the quadruplex folding of the KRAS promoter sequence. Investigations of the GQ^KRAS^/HMGB1 co-condensates indicate that the ssDNA of the KRAS promoter retains quadruplex folding in the droplets, while the droplets formed by GQ^KRAS^/HMGB1 are in liquid-like phase and rather dynamic. The quadruplex-folding of the KRAS promoter is required for the co-condensation with HMGB1. Mechanistic investigations reveal that, in addition to electrostatic interactions, the recognition of GQ^KRAS^/HMGB1 also plays key roles in the co-condensation of two macro-biomolecules; while hydrophobic interactions are not involved in the LLPS of these highly hydrophilic molecules. This result indicates that the well-folded proteins could modulate the structure and function of GQ DNA through phase separation in a mutual promotion manner.

## Supplementary Material

gkad938_supplemental_files

## Data Availability

The data underlying this article are available in the article and in its online supplementary material.
