# Supplementary material for: Mutual promotion of co-condensation of KRAS G-quadruplex and a well-folded protein HMGB1
Source: Nucleic Acids Res. 2023 Oct 28;52(1):288–99. doi: 10.1093/nar/gkad938 (PMC10783520; doi:10.1093/nar/gkad938)
Supplement: gkad938_supplemental_files [file gkad938_supplemental_files.zip › SI-NAR_LLPS_09_07.pdf]

SUPPLEMENTARY DATA

Table S1. DNA sequence used in the experiments

Oligonucleotides abbreviation	Sequence (5'–3')
22R	AGGGCGGTGTGGGAAGAGGGAA
m22R	AAAACAATATAAAAAGAAAAAA
s22R	GAGGCGGTGTGGAGAGAGAGAG
LTRIII	GGGAGGCGTGGCCTGGGCGGGACTGGGG
2G	TGCCATTGGTTGGTGTGGTTGG
h-Tel	GGGTTAGGGTTAGGGTTAGGG
Random	GAATCGACAGTATGCGATAACT
F-22R-T	6-FAM-AGGGCGGTGTGGGAAGAGGGAA-TAMRA

Table S2. Primers used for constructing protein mutants

Primers	Sequence (5'–3')
H31A_Forward	CATAAGAAGAAGGCCCCAGATGCTTCA
H31A_Reverse	TGAAGCATCTGGGGCCTTCTTCTTATG
K44A_Forward	GAGTTTTCTAAGGCGTGCTCAGAGAGG
K44A_Reverse	CCTCTCTGAGCACGCCTTAGAAAACTC
K50A_Forward	TCAGAGAGGTGGGCGACCATGTCTGCT
K50A_Reverse	AGCAGACATGGTCGCCCACCTCTCTGA
K65A_Forward	GAAGATATGGCAGCAGCGGACAAGGCC
K65A_Reverse	GGCCTTGTCCGCTGCTGCCATATCTTC
R70A_Forward	GCGGACAAGGCCGCTTATGAAAGAGAA
R70A_Reverse	TTCTCTTTCATAAGCGGCCTTGTCCGC
Y71A_Forward	GACAAGGCCCGTGCTGAAAGAGAAATG
Y71A_Reverse	CATTTCTCTTTCAGCACGGGCCTTGTC

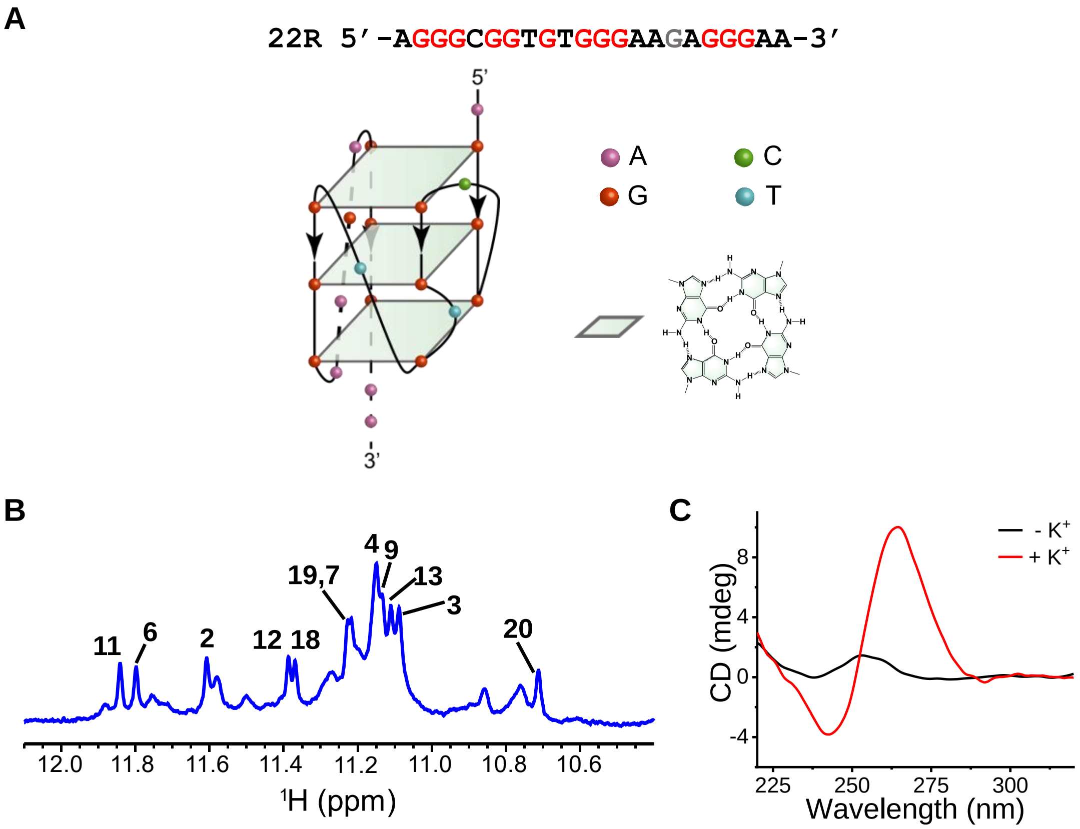

Figure S1. Formation of G-quadruplex of 22R sequence. (A) The sequence of human 22R (upper panel) used in this work and schematic illustration of the GQ^{KRAS} folding (lower panel). (B) ^{1}H-NMR spectrum of 100 μM 22R in potassium phosphate buffer (pH = 7.4) containing 120 mM K^+. The spectrum was recorded at 25°C. (C) Circular dichroism (CD) spectra of 10 μM 22R in H_2O (black) or potassium phosphate buffer (pH = 7.4) containing 100 mM K^+ (red).

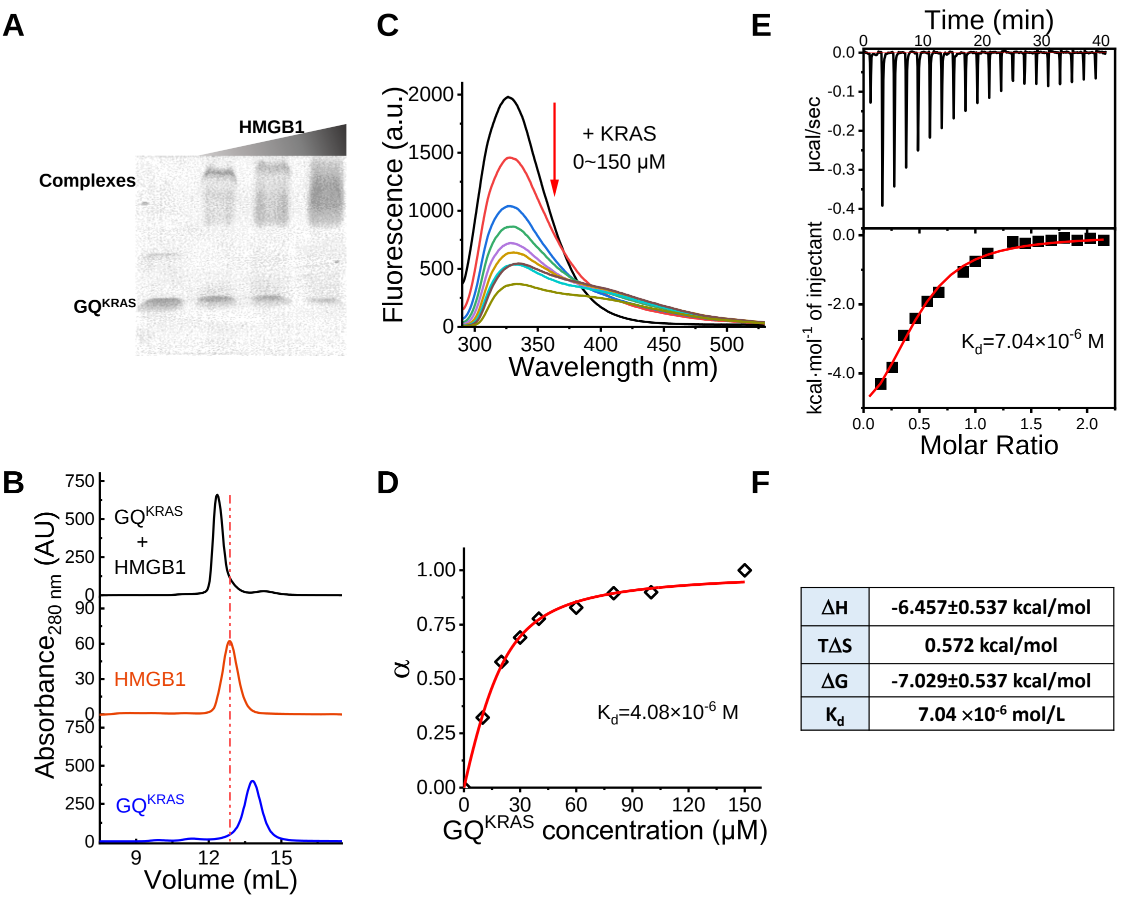

ΔH	-6.457±0.537 kcal/mol
TΔS	0.572 kcal/mol
ΔG	-7.029±0.537 kcal/mol
K_d	7.04 ×10^{-6} mol/L

Figure S2. Characterization of interaction of GQ^{KRAS} and HMGB1. (A) Electrophoretic mobility shift assay (EMSA) for assessing the binding of HMGB1 to GQ^{KRAS} (50 μM). (B) Size exclusion chromatography (SEC) analysis of GQ^{KRAS}/HMGB1 complex formation. (C) Fluorescence titration of GQ^{KRAS} (0 – 150 μM) to HMGB1 (10 μM) at 25°C. The spectra were recorded with excitation at 280 nm. (D) Plot of the fraction of GQ^{KRAS}-bound HMGB1 (α) during the titration of GQ^{KRAS}. The experimental data (black squares) were calculated based on the result of Figure S2C, and the fitting curve was obtained using independent and equivalent-sites model. (E) Isothermal titration calorimetry (ITC) analysis of the interaction of HMGB1 with GQ^{KRAS}. Raw data were obtained by titration of GQ^{KRAS} (400 μM) into HMGB1 (40 μM, 400 μL) at 25°C (upper panel), and the data were fitted with the Origin one-site model (lower panel). (F) The thermodynamic parameters obtained from ITC fitting in Figure S2E.

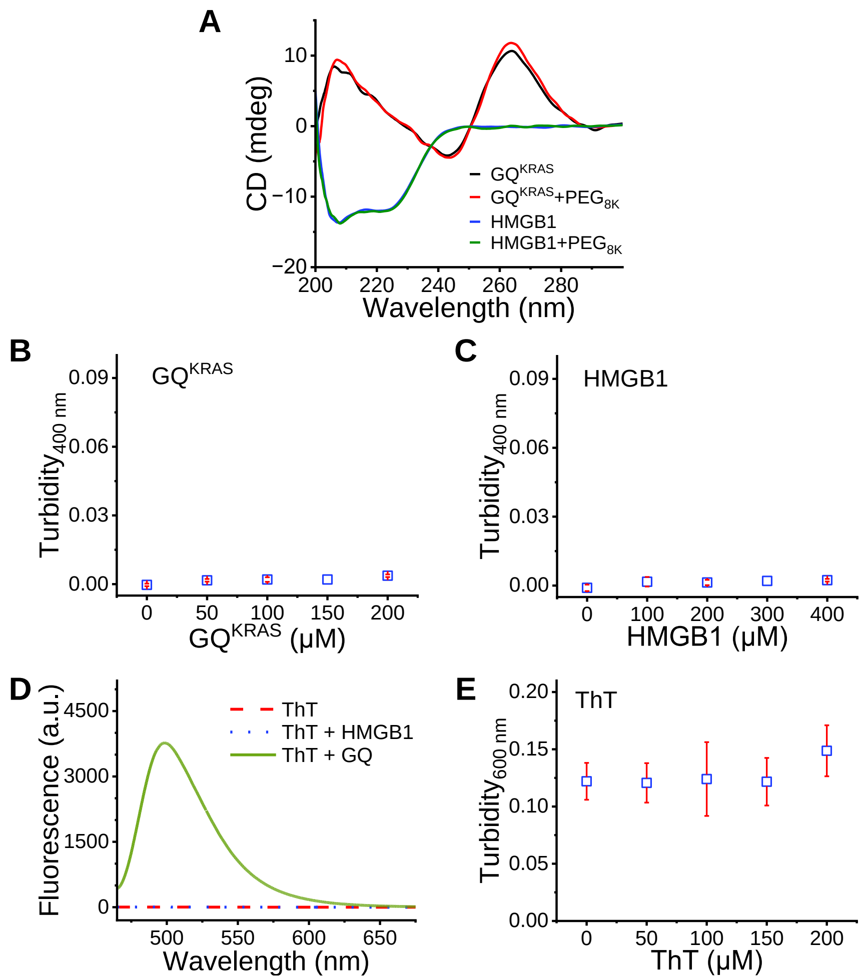

Figure S3. (A) CD spectroscopic analysis of the effect of PEG_{8K} on the folding of GQ^{KRAS} and HMGB1. Experiments were performed on GQ^{KRAS} (10 μM) or HMGB1 (10 μM) in 10 mM HEPES (containing 20 mM K^+) in the presence or absence 10% PEG_{8K}. (B) Turbidity of GQ^{KRAS} alone at different concentrations. (C) Turbidity of HMGB1 alone at different concentrations. (D) Fluorescence spectra of ThT in the absence (red dashed line) or the presence of HMGB1 (blue dotted line), GQ^{KRAS} (green solid line) in 10 mM HEPES buffer (pH 7.4) containing 20 mM K^+. (E) Effect of ThT on the co-condensation of GQ^{KRAS} (25 μM) and HMGB1 (50 μM). Samples were prepared in 10 mM HEPES buffer (pH 7.4) containing 20 mM K^+ and 10% PEG_{8K}.

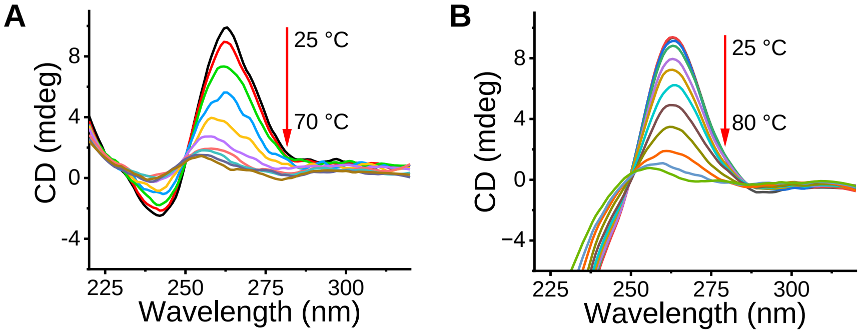

Figure S4. CD melting analysis of the effect of HMGB1 on the GQ^{KRAS} folding. CD spectra were recorded on 10 μM GQ^{KRAS} in the absence (A) or presence (B) of 30 μM HMGB1 in potassium phosphate buffer (pH = 7.4) containing 120 mM K^+ at different temperatures (from 25 to 80°C with an increase of 5°C).

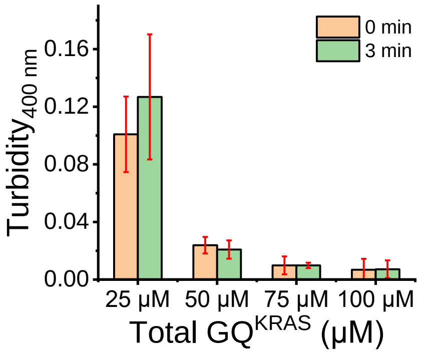

Figure S5. Turbidity analysis of the effect of excessive GQ^{KRAS} on the co-condensation of GQ^{KRAS}/HMGB1. The phase-separation samples were prepared using GQ^{KRAS} (25 μM) with HMGB1 (50 μM) in 10 mM HEPES buffer (pH 7.4) containing 20 mM K^+ and 10% PEG_{8K}. The turbidity was measured immediately after adding GQ^{KRAS} (orange columns) and measured again after 3 minutes (green columns).

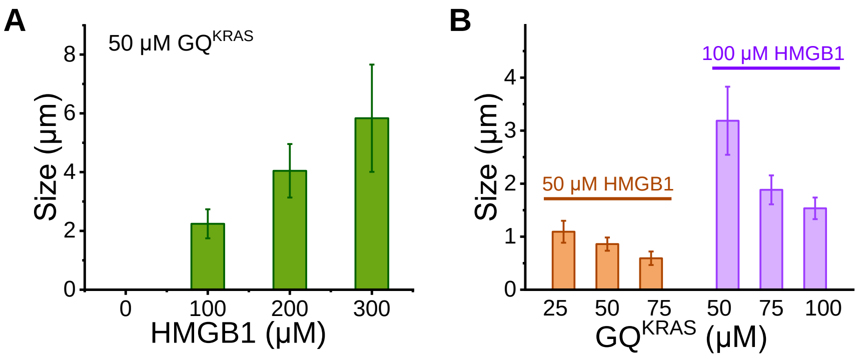

Figure S6. Sizes of droplets observed on fluorescence images in Figure 4C and 4D. (A) Droplets formed by GQ^{KRAS} (50 μM) with different concentration of HMGB1. (B) Droplets formed by HMGB1 (50 μM or 100 μM) with different concentrations of GQ^{KRAS}. The droplet sizes were assessed on ten representative droplets in samples. Too large and too small droplets were not included. Graphs are presented as mean ± sd.

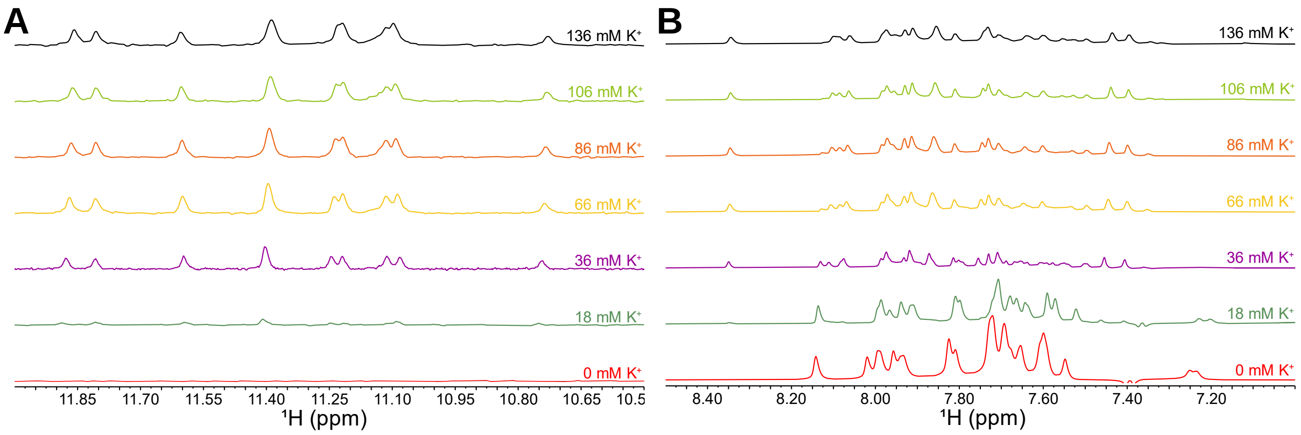

Figure S7. ^{1}H-NMR spectra of 22R DNA in different concentration of K^{+} ions. (A) Selected region shows the signal of imino protons. (B) Selected region shows signal of aromatic protons. The spectra were recorded on 0.5 mM 22R DNA at 25°C in potassium phosphate buffer (pH 6.5) containing different K^{+} concentrations as indicated.

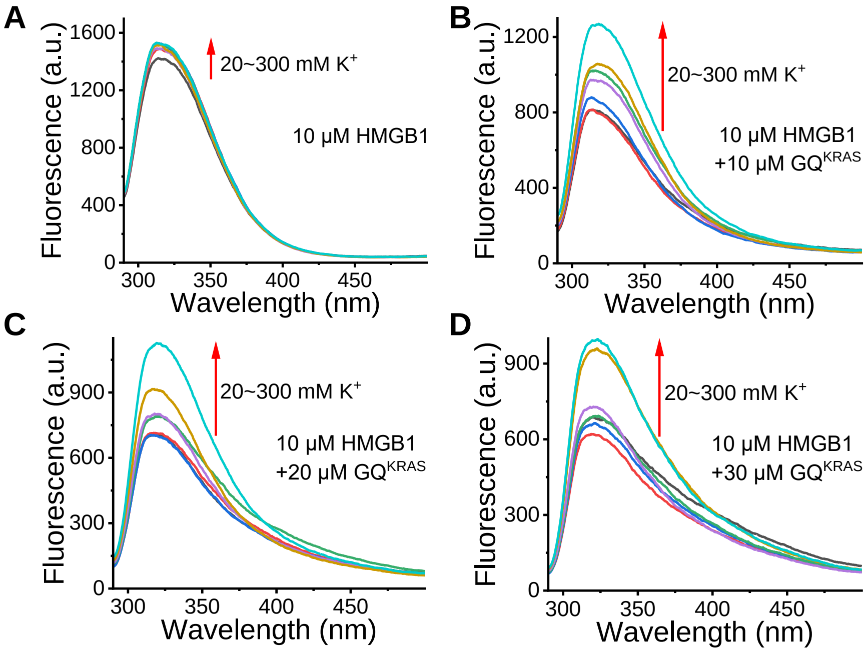

Figure S8. Effect of K^+ ions on the interaction of HMGB1 and GQ^{KRAS}. K^+ ions (20 – 300 mM) were added to the HMGB1 (10 μM) containing different concentrations of GQ^{KRAS} in 10 mM HEPES buffer (pH 7.4). (A) 0 μM GQ^{KRAS}; (B) 10 μM GQ^{KRAS}; (C) 20 μM GQ^{KRAS}; (D) 30 μM GQ^{KRAS}.

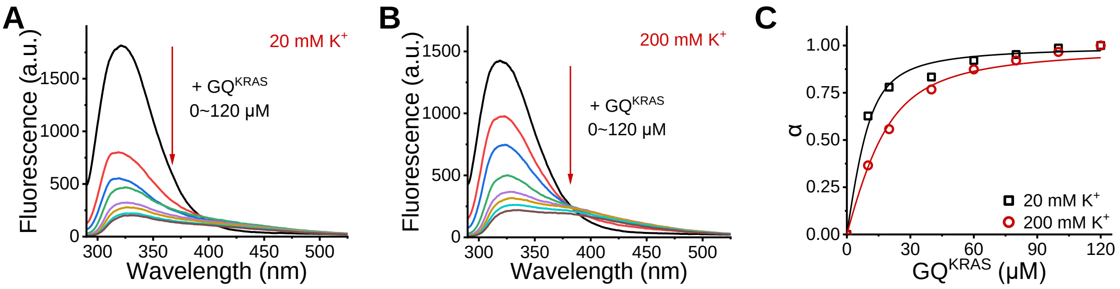

Figure S9. Analysis of the binding affinity of GQ^{KRAS} to HMGB1 through fluorescence titration. Fluorescence spectra of HMGB1 (10 μM) were recorded in the presence of different concentration of GQ^{KRAS} (0-120 μM) in 10 mM HEPES buffer (pH 7.4) containing 20 mM K^+ (A) or 200 mM K^+ (B). (C) Fitting titration data obtained from spectra in (A) and (B). The fractions of GQ^{KRAS}-bound HMGB1 (α) were calculated based on the fluorescence intensity changes during titration.

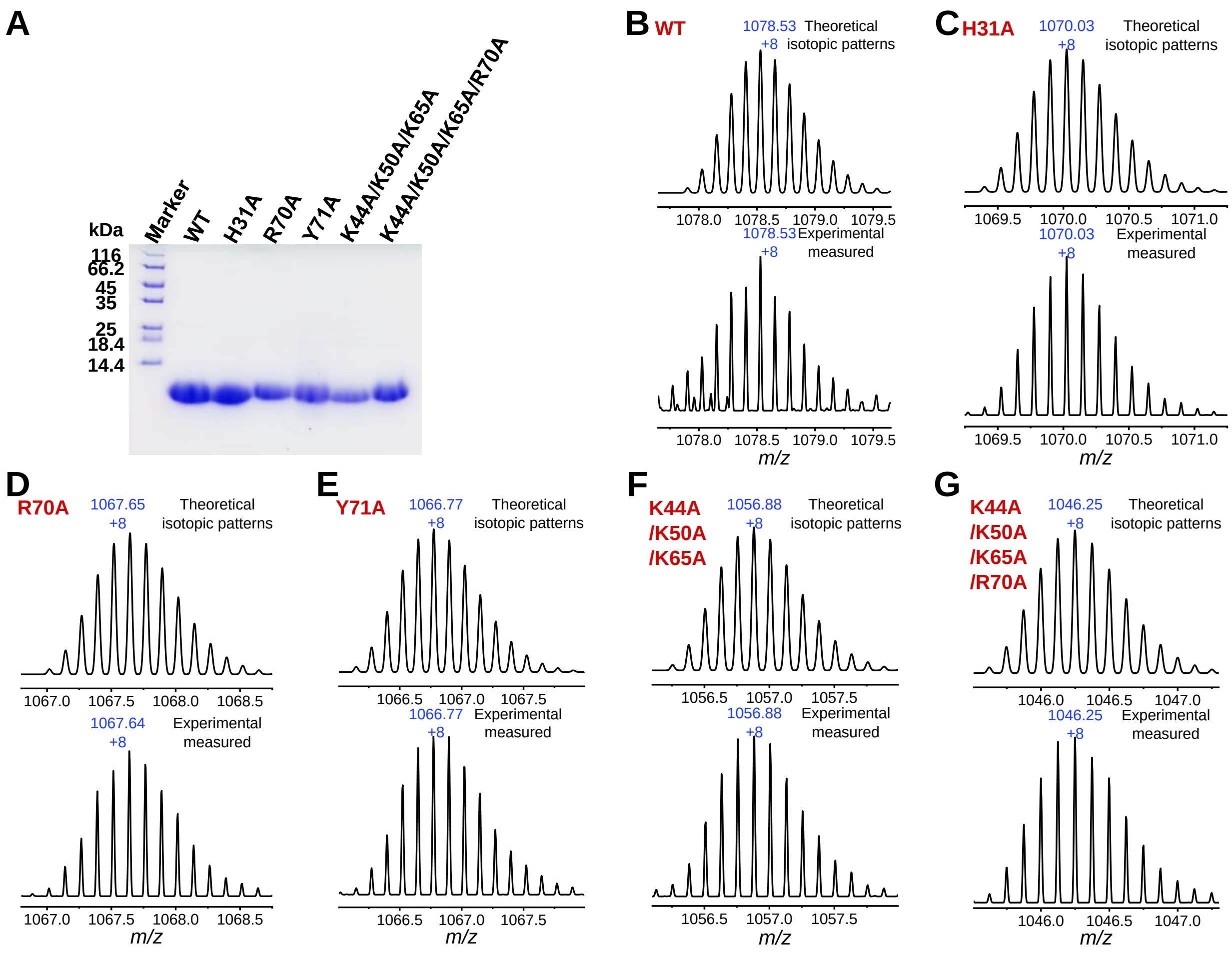

Figure S10. (A) Tricine-SDS-PAGE to assess the purities of HMGB1 and its mutated proteins. (B-G) ESI-MS spectra of HMGB1 and its mutated proteins as indicated in figures. The mutation sites are indicated in figures. 8+ charged peaks and their theoretical isotopic patterns are shown.

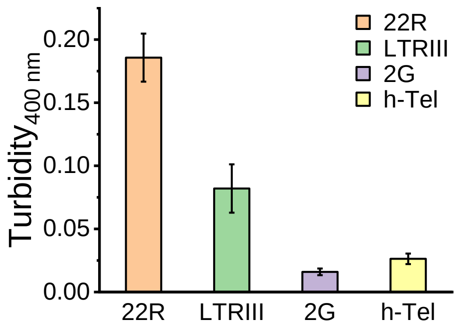

Figure S11. The turbidity analysis of other GQs (50 μM, listed in Table S1) after incubation with HMGB1 (75 μM) in 10 mM HEPES buffer (pH 7.4) containing 20 mM K^+ and 10% PEG_{8K}.

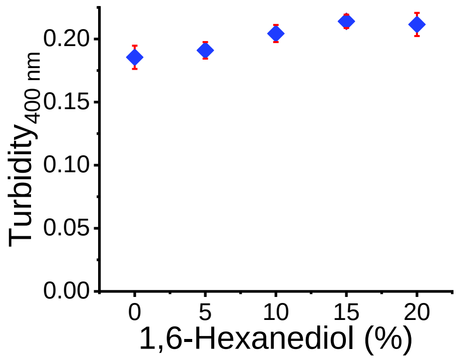

Figure S12. Effects of 1,6-hexanediol on the co-condensation of GQ^{KRAS}/HMGB1. 0 – 20% 1,6-hexanediol was added to the phase-separation sample prepared by 50 μM GQ^{KRAS} and 75 μM HMGB1 in 10 mM HEPES buffer (pH 7.4) containing 20 mM K^+ and 10% PEG_{8K}.

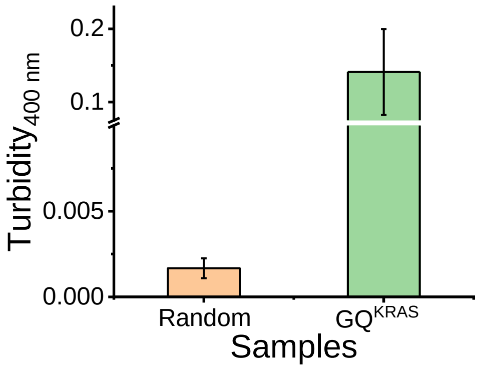

Figure S13. Comparison of different DNA sequence on the co-condensation of GQ^{KRAS}/HMGB1. Turbidity analysis of 75 μM HMGB1 with 50 μM DNA in 10 mM HEPES buffer (pH 7.4) containing 20 mM K^+ and 10% PEG_{8K}. Random denotes a DNA sequence that can not form quadruplex.

Video S1. Fluorescence recovery after photobleaching experiments within the GQ^{KRAS}/HMGB1 droplets in 10 mM HEPES buffer (pH 7.4) containing 20 mM K^+ and 10% PEG_{8K}. See the video in a separated file.

Video S2. Growth and fusion of GQ^{KRAS}/HMGB1 droplets. See the video in a separated file.
